# Experimental Investigation of Wave Propagation Characteristics in Entangled Metallic Wire Materials by Acoustic Emission

**DOI:** 10.3390/ma16134723

**Published:** 2023-06-29

**Authors:** Yanhong Ma, Tianyu Liang, Yongfeng Wang, Qicheng Zhang, Jie Hong

**Affiliations:** 1 Research Institute of Aero-Engine, Beihang University, Beijing 100191, China; 2 School of Energy and Power Engineering, Beihang University, Beijing 100191, China; 3 Faculty of Science and Engineering, Swansea University, Swansea SA1 8EN, UK

**Keywords:** entangled metallic wire material, wave propagation, acoustic emission, response characteristics, parameter analysis, acoustic frequency vibration

## Abstract

In this paper, the response characteristics of wave propagation in entangled metallic wire materials (EMWMs) are investigated by acoustic emission. The frequency, amplitude of wave emission, and the pre-compression force of the specimen can be adjusted in the experimental setup. EMWM specimens fabricated from stainless steel wires and with different design parameters are tested in this work. The results show that waves of different amplitudes propagate in EMWMs with approximate linear characteristics and the fluctuation coefficient of wave passing ratios is calculated below 15%. The response spectrum of passing waves shows a distinct single-peak characteristic, with the peak response at approximately 14 kHz. The parameters of pre-compression force, porosity, wire diameter, helix diameter, specimen height, and the layered structure of specimens have no significant effect on the frequency characteristics but moderately affect the wave passing ratios. Notably, EMWMs exhibit a lower wave passing ratio (ranging from 0.01 to 0.18) compared to aluminum alloy and natural rubber. The characteristics of response spectrums can be successfully reproduced by the finite element simulation. This work demonstrates EMWMs’ potential as an acoustic frequency vibration isolation material, offering excellent performance and engineering design convenience.

## 1. Introduction

Entangled metallic wire material (EMWM) is a sort of functional porous material created through a multi-step fabrication process involving winding, stretching, weaving, molding, and post-treatment of metallic wires. The interconnected arrangement of coiled wires results in the formation of gaps and holes, contributing to the material’s lightweight characteristic. Under various loading conditions, the coiled wires undergo elastic or plastic deformation, resulting in changes in contact status and the occurrence of micro-slips at the engagement points. These mechanical behaviors lead to the high elasticity and damping properties of EMWMs [1]. The performance of EMWMs can be designed by manufacturing parameters. With a simple manufacturing process, it has been widely applied in engineering fields such as vibration isolation [2,3], energy absorption [4], seal filtering [5,6], and biological implantation [7]. Consequently, the mechanical characteristics of EMWMs under different loads attract the research interest of numerous scholars. The static mechanical characteristics of EMWMs have been extensively studied, including analyses of their structural organization [8], mechanical experiments [9,10,11], and constitutive model investigations [12,13]. In terms of dynamic characteristics, the literature primarily focuses on the study of low-frequency vibrations (0–500 Hz) [14,15] and energy absorption properties [16,17]. There is a lack of literature reporting on the mechanical characteristics of EMWMs in a higher frequency range (up to the level of several kHz), which limits their application in the field of acoustic frequency mechanical vibration isolation. For instance, the authors of this paper attempt to apply EMWM solids as a sound-absorbing material to reduce aerodynamic noise from the mechanical structures in a jet aircraft. The target frequency for this application ranges from 5 to 20 kHz.

The primary task in applying EMWMs to acoustic frequency vibration isolation is to study the propagation characteristics of waves in this material. For other types of porous materials, such as honeycomb structures [18,19], foams [20,21], woven fibers [22,23], and cellular materials [24], there is a wealth of research on the wave propagation. For periodic structural materials, wave and finite element methods have been developed to study the frequency band characteristics of wave propagation throughout the entire structure. By solving the representative unit cell models and applying the principle of periodic extension, the frequency band characteristics of wave propagation can be obtained [25,26,27]. For non-periodic materials, such as foams, structural generation algorithms can be conveniently used to obtain finite element models based on parameter constraints [28,29]. In contrast, the meso-scale structure of EMWMs is complex, with numerous influencing parameters, making it challenging to establish finite element models. Hence, utilizing experimental techniques to explore the characteristics of wave propagation is a good starting point for research.

Acoustic emission refers to the phenomenon where a localized deformation occurs in a component or material subjected to external forces, resulting in the rapid release of energy and the generation of transient waves. Derived from this phenomenon, the acoustic emission non-destructive testing technique can be applied to detect and locate manufacturing defects, metal fatigue, and internal damage or failure in structures. Engineers often refer to it metaphorically as the ‘stethoscope’ for materials [30,31]. The working principle involves the propagation of elastic waves emitted from the acoustic emission source to the surface of the material, causing displacement on the surface of the acoustic emission sensor. The mechanical vibrations of the material are then converted into electrical signals through piezoelectric crystals. These signals are subsequently amplified, recorded, and processed. The waves collected can be utilized for waveform and spectrum analysis. Some researchers also use mode recognition algorithms to analyze the characteristics of defects and determine their locations within the structures [32,33].

This paper utilizes the capability of the acoustic emission sensor which can both emit and receive high-frequency waves. An experimental system is designed to study the wave propagation characteristics in EMWMs within the set acoustic frequency range (0–20 kHz) and under the influence of parameters. This paper is organized into six sections. Section 1 is the introduction. Section 2 briefly introduces the EMWM specimens used in the experiment and the process parameters considered, including porosity, wire diameter, helix diameter, specimen height, and layered structure. Section 3 introduces the acoustic emission experimental equipment and specimen clamping structure, the wave propagation characteristics of the acoustic emission sensor, and the calculation method for the wave passing ratio of the wave propagation through the EMWM specimens. Section 4 presents the experimental results and discussions, including the linear characteristics between the wave passing ratio and the input voltage, as well as the frequency response characteristics of the EMWM specimens. Section 5 presents the establishment of finite element models for the material specimens using computed tomography scanning and compares the numerical simulation results with the experimental results to validate the wave propagation characteristics. Section 6 concludes the paper, summarizing the response characteristics of wave propagation in EMWMs obtained by acoustic emission.

## 2. EMWM Specimens

**Figure 1:**
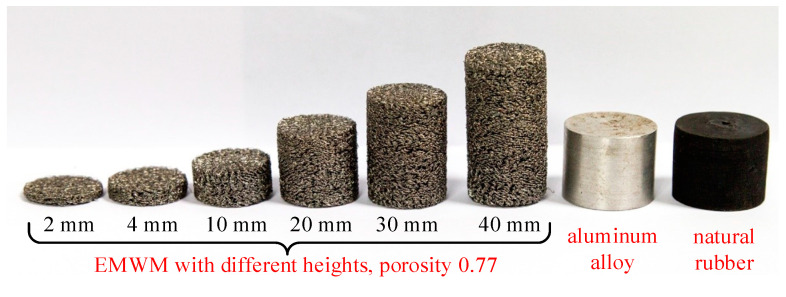
Specimens of EMWMs, aluminum alloy, and natural rubber.

The raw material wires for the EMWM specimens are provided by Anping Risheng Wire Mesh Ltd., in Hengshui, China. The grade of stainless steel wires used is 0Cr18Ni9. Firstly, the steel wires are tightly wound into closely spaced helixes using a wire winding machine. Then, the helixes are stretched according to the designed pitch to form coiled wires. These coiled wires are then woven into a blank in the weaving process. Finally, the blank is cold-pressed into the desired cylindrical shape of EMWM specimens using molds and a press device. The specimens have a specified diameter of 20 mm, which is consistent with the acoustic emission sensor, and the height can be adjusted. The EMWM specimens in this work are divided into five groups to investigate the effects of porosity, wire diameter, helix diameter, specimen height, and layered structure on the wave propagation characteristics. Some of the specimens used in the experiment are shown in Figure 1. The parameters of EMWM specimens tested in this work are shown in Table A1 in Appendix A.

Porosity: For EMWM specimens, the primary design parameter that influences the mechanical properties is porosity ϕ. Porosity describes the volume fraction of gaps between the coiled wires and it can be defined as Equation (1). Some of the literature also uses relative density to characterize the porosity of EMWM.

(1) $$\phi =1-\frac{V_{w}}{V_{s}}=1-\frac{M_{s}}{\rho_{w}V_{s}}$$

where $V_{w}$ is the volume of coiled wires. $V_{s}$ is the total volume of the specimen. $M_{s}$ is the total mass of the specimen. $\rho_{w}$ is the density of coiled wires.

**Figure 2:**
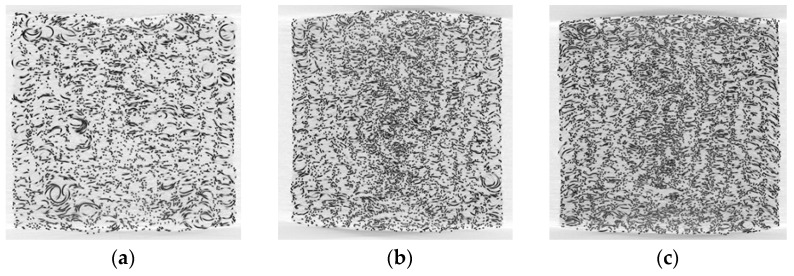
CT scanning images of cross-section in EMWM specimens with different porosities of (**a**) 0.83, (**b**) 0.77, and (**c**) 0.72.

Porosity of the specimen can be adjusted by controlling the mass of the wire used. In this work, EMWM specimens with a porosity of 0.83, 0.77, and 0.72 are fabricated. These porosity values cover a range suitable for engineering applications in vibration isolation and noise reduction. The cross-sectional images of the specimens obtained through computed tomography (CT) scanning are shown Figure 2. These images clearly depict the varying arrangement and tightness of the coiled wires, which directly reflect the stiffness characteristics subjected to a load.

Wire diameter: The wire diameter refers to the diameter d of the steel wires used to construct the EMWM. In this work, wire diameters of 0.08 mm, 0.15 mm, and 0.25 mm are tested.

Helix diameter: The helix diameter refers to the outer diameter of the helical wires produced by the wire winding machine. In this work, helix diameters of 1 mm, 1.5 mm, and 2 mm are tested.

Specimen height: The specimen height refers to the vertical dimension of the cylindrical specimens, which are produced with a diameter of 20 mm. In this work, specimen heights of 2 mm, 4 mm, 10 mm, 20 mm, 30 mm, and 40 mm are tested.

Layered structure: The existence of a boundary in EMWMs, where the arrangement of the coiled wires is looser compared to the inner layers, has a significant influence on the mechanical properties [34]. The smaller the height of the specimen, the larger the volume fraction of the boundary layer within the EMWM, resulting in a decrease in quasi-static modulus and an increase in the damping factor. To investigate the effect of the boundary, thin specimens with the same parameters and a height of 10 mm are connected in series. The layered structure is achieved by placing a 0.2 mm aluminum spacer between each specimen.

In addition to the EMWM specimens mentioned above, for comparison purposes, cylindrical samples with a diameter of 20 mm and a height of 20 mm are prepared using aluminum alloy (grade 2A12) and natural rubber.

## 3. Experimental Methods

### 3.1. Experimental Setup

**Figure 3:**
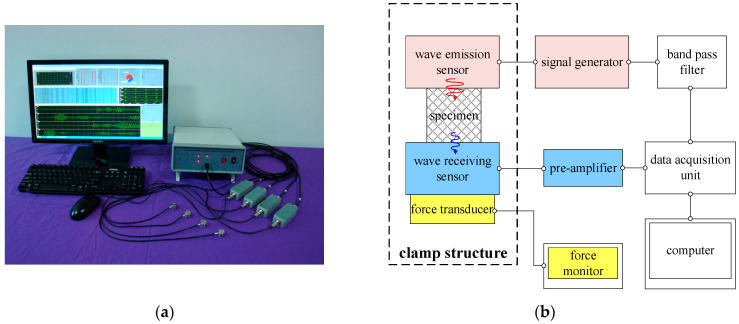
(**a**) Acoustic emission equipment used in the work; (**b**) schematic diagram of the experimental setup.

The experimental setup uses the DS2 type Acoustic Emission Signal Analyzer produced by Beijing Ruandao Technology Co., Ltd., Beijing, China. The photograph of the equipment can be seen in Figure 3a. The working principle of the acoustic emission sensor is based on the positive piezoelectric effect of the piezoelectric ceramic plate, which converts mechanical vibrations into voltage signals for data acquisition. The sensor (RS-2A type, produced by Beijing Ruandao Technology Co., Ltd.) is capable of receiving frequencies in the range of 1 kHz to 1 MHz. By utilizing the inverse piezoelectric effect, the acoustic emission sensor can also be used as an exciter or actuator. By inputting an alternating voltage signal to the sensor, the dielectric of the piezoelectric ceramic plate undergoes synchronized polarization and deformation under the electric field, resulting in acoustic frequency mechanical vibrations as the excitation input. In the wave propagation experiment, two identical acoustic emission sensors are used, one as the excitation source to emit waves, and the other as the receiver to capture the wave propagation through the EMWM specimen. Based on the aforementioned experimental principle, the wave propagation experimental system designed in this work is shown in Figure 3b.

**Figure 4:**
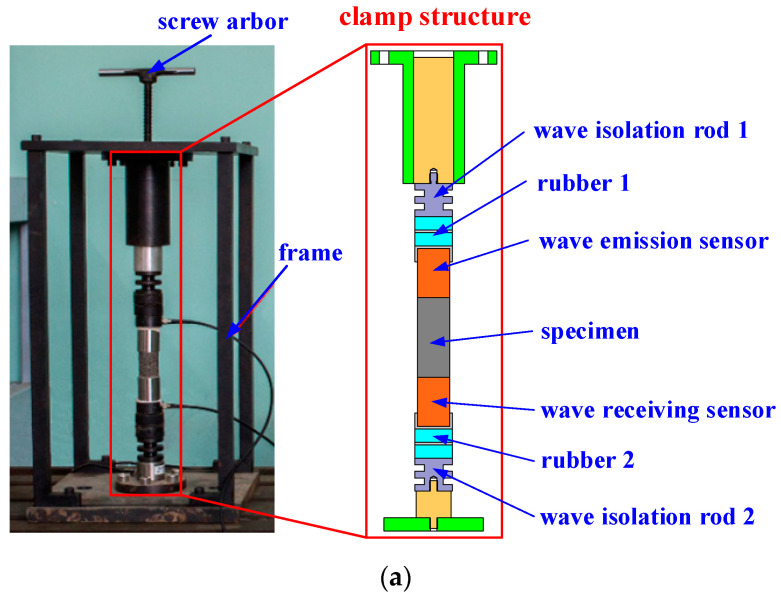

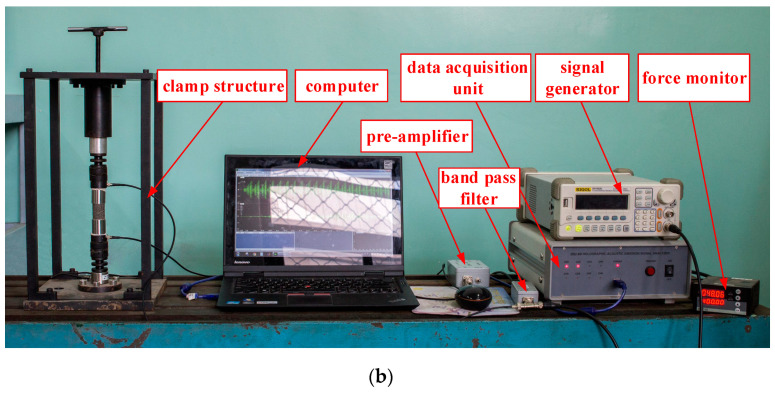
(**a**) Clamping structure of the specimens; (**b**) photograph of the acoustic emission experimental setup.

A clamping structure adjusting the pre-compression force of the specimen is designed in this work, as shown in Figure 4. The components of the clamping structure from top to bottom are as follows: compression screw, pressure rod, isolation rod 1, isolation rubber 1, wave emission sensor, specimen, wave receiving sensor, isolation rubber 2, isolation rod 2, and force transducer. Two acoustic emission sensors are placed in close proximity to the ends of the specimen. One sensor serves as the wave emission sensor and is connected to the signal generator, while the other sensor serves as the wave receiving sensor and is connected to the data acquisition unit via a signal amplifier. When adjusting the pre-compression force, the screw is tightened to move the pressure rod downward. The compression force is transmitted through the isolation rod 1, isolation rubber 1, and the wave emission sensor onto the specimen. The magnitude of the pre-compression force is measured by the force transducer. In the experiment, pre-compression forces of 50 N, 100 N, 150 N, 200 N, 250 N, and 300 N are applied to the specimen. To prevent multiple reflections of waves within the clamping structure from interfering with the experimental results, multiple layers of isolation rubbers and isolation rods with section geometry variations are designed. This arrangement can effectively isolate interference waves.

**Figure 5:**
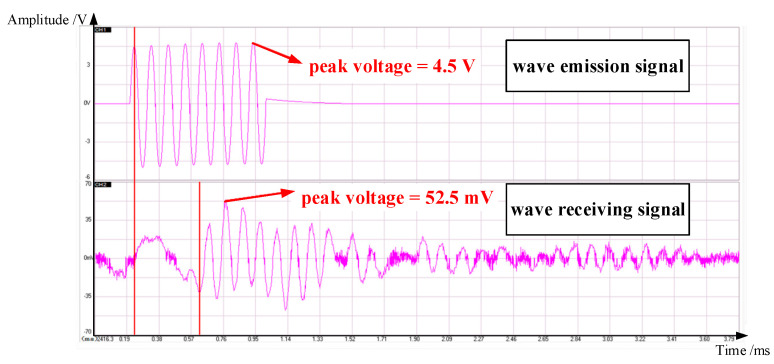
Time-domain signals at the emission and receiving ends of acoustic emission equipment.

In the experiment, the emission wave is an 8-wavelength sinusoidal signal. The frequency of the emission wave is determined based on the experimental frequency, ranging from 0 to 20 kHz, and remains the same with each emission. It is transmitted every 100 ms, and the receiving end detected the transmitted wave with a certain time delay. The received signal largely preserves the waveform of the emission signal. An example of the time-domain signal for one typical emission and reception is shown in Figure 5. In order to account for the varying amplitude of the receiving waves, this work defines the average voltage amplitude of the receiving waves (each parameter combination tested three times) by extracting the peak values of the first four complete waveforms and taking their average ${\bar{U}}_{tr}$. Following the same rules, the average voltage amplitude ${\bar{U}}_{in}$ of the emission waves is calculated. Due to the excellent reproducibility of the experimental data, no data was discarded.

Based on the approximate proportionality between the measured vibration displacements by the voltage value of the emission sensor (an accelerometer has been used to preliminarily validate this relationship), the expression for calculating the wave passing ratio $r_{p}$ is as follows:

(2) $$r_{p}=\frac{A_{tr}}{A_{in}}=\frac{{\bar{U}}_{tr}}{{\bar{U}}_{in}}$$

where $A_{in}$ and $A_{tr}$ are the vibration displacement amplitudes at the wave incident end and wave exit end of the specimen, respectively.

To ensure the accuracy of the experimental results, the average of the three measured wave passing ratios $r_{p}$, obtained from three sets of emission and reception measurements, is considered as the final experimental result.

### 3.2. Characteristics of Acoustic Emission Sensor

Before the formal experiment, the two acoustic emission sensors are brought into direct contact and applied with different pre-compressive forces and input voltages in order to study the wave emission and reception characteristics of the sensors.

**Figure 6:**
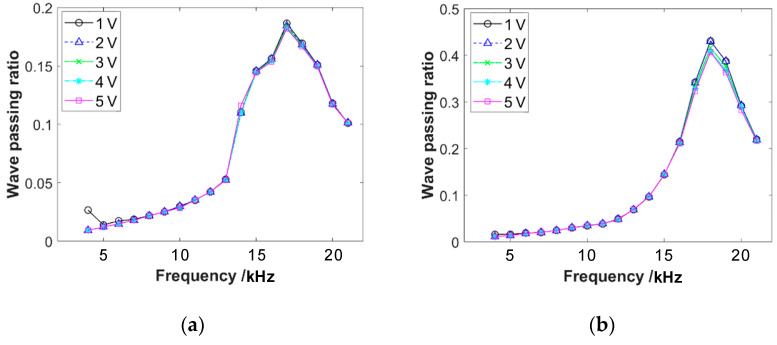
The frequency response curves of wave propagation through two acoustic emission sensors under different input voltages, with the pre-compression force of (**a**) 50 N and (**b**) 150 N.

With pre-compressive forces of 50 N and 150 N, respectively, the emission sensor is subjected to different input voltages, resulting in emission waves of varying amplitudes. The frequency response curves of the wave propagation through the sensor are shown in Figure 6. The graphs reveal that the sensor causes significant attenuation of the wave propagation. The frequency response curve exhibits a distinct single-peak characteristic, with the peak frequency ranging from 15 kHz to 20 kHz. This result will greatly impact the measurement of wave passing ratios through the specimen. The response characteristics of the sensor exhibit approximately linear characteristics within the input voltage range of 1–5 V. That is in accordance with the experimental requirements.

**Figure 7:**
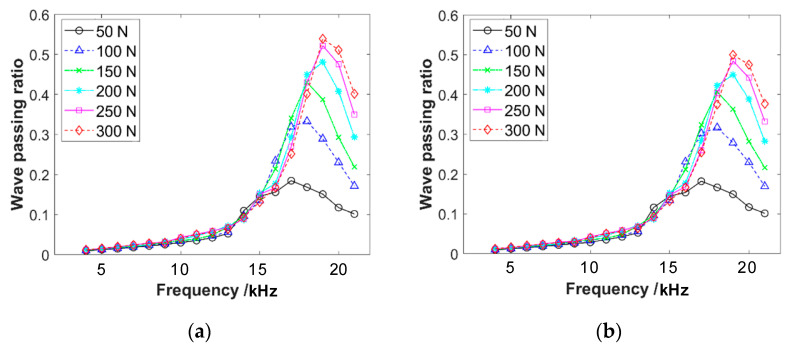
The frequency response curves of wave propagation through two acoustic emission sensors under different pre-compression forces, with the input voltages of (**a**) 2 V and (**b**) 5 V. These two voltage values are sufficiently representative for the range of 1–5 V.

With input voltages of 2 V and 5 V, respectively, different pre-compressive forces are applied to the acoustic emission sensors and the frequency response curves are obtained, as shown in Figure 7. It can be observed that the peak of wave passing ratios increases with the increase in pre-compressive force. Additionally, the single peak shifts towards higher frequencies. This result will also affect the measurement of the response of wave propagation through the specimen corresponding to different pre-compressive forces.

### 3.3. Data Processing

To eliminate the influence of the response characteristics of the acoustic emission sensor on the measurement results of the specimen, the normalized wave passing ratio $r_{p,n}$ is defined in this work, which is obtained by dividing the wave passing ratio $r_{p}$ measured from the specimen by the wave passing ratio r of the acoustic emission sensor under the same experimental conditions (pre-compressive force, voltage, frequency). The expression is as follows:

(3) $$r_{p,n}=\frac{r_{p}}{r}$$

## 4. Results and Discussion

### 4.1. Linear Characteristics

Input voltages of 1 V, 2 V, 3 V, 4 V, and 5 V are applied to the wave emission sensor in the experiment. Because the excitation amplitude generated by the sensor is directly proportional to the input voltage, it enables the study of the response characteristics of wave propagation through EMWMs with different excitation amplitudes.

**Figure 8:**
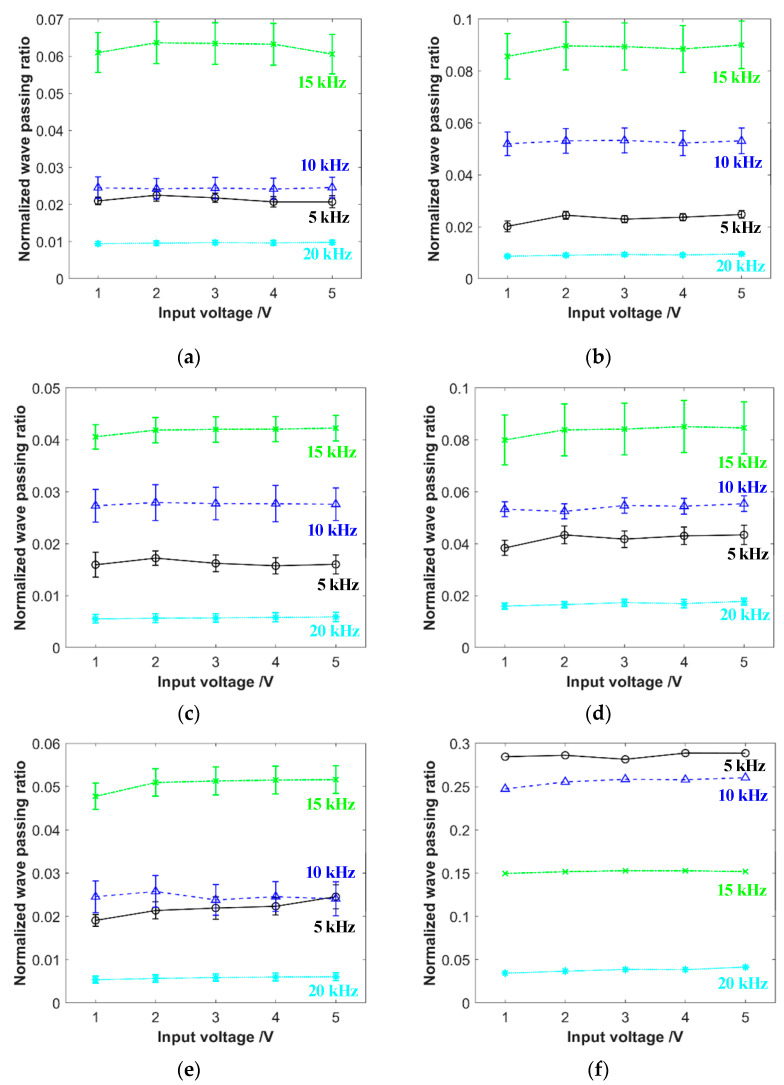
The influence curves of different input voltages on wave passing ratio, (**a**) specimen no. 1, with the pre-compression force of 50 N; (**b**) specimen no. 2, with the pre-compression force of 100 N; (**c**) specimen no. 9, with the pre-compression force of 150 N; (**d**) specimen no. 3, with the pre-compression force of 300 N; (**e**) double-layered specimen, with the pre-compression force of 200 N; (**f**) natural rubber specimen, with the pre-compression force of 250 N.

The results of the wave passing ratio for some certain specimens under different input voltages, excitation frequencies, and pre-compression forces are shown in Figure 8. The calculated fluctuation amplitude of the wave passing ratios for the EMWM specimens is found to be less than 15%. The response does not exhibit a monotonic behavior with increasing voltage. Therefore, it can be concluded that the fluctuation in the response is within the range of experimental error. This result indicates that the wave propagation characteristics of the EMWM exhibit good linearity, which means that the wave passing ratio is independent of the excitation amplitude.

The reason for the linear characteristics is because, in the experiments, the applied excitation amplitudes are small and the excitation frequencies are high. In the acoustic frequency and small amplitude vibration conditions, the coiled wires inside EMWMs are hooked with each other, and a large number of contact points are primarily in a state of sticking. Only a small part may undergo slight slipping. As a result, the mechanical characteristics of EMWMs remain largely unchanged under this loading condition.

### 4.2. Frequency Response Characteristics

**Figure 9:**
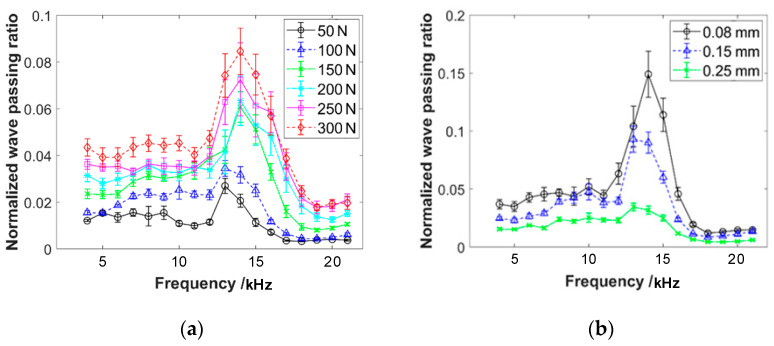

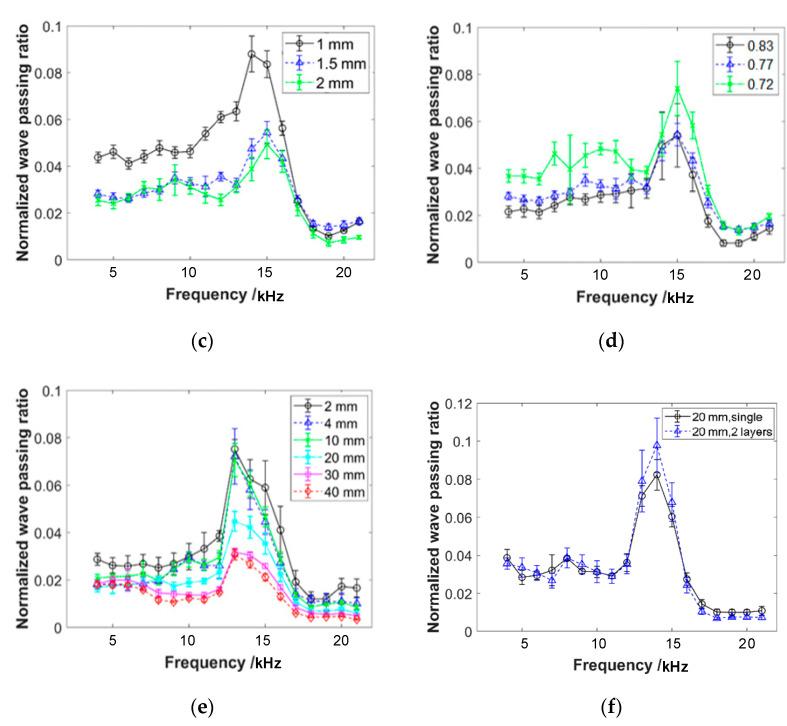
The frequency response curves of wave propagation of specimens with different parameters and experimental conditions of (**a**) pre-compression force, (**b**) wire diameter, (**c**) helix diameter, (**d**) porosity, (**e**) specimen height, and (**f**) double-layered specimen.

The experiment applies wave excitation to specimens with different pre-compression forces, porosity, wire diameter, helix diameter, specimen height, and layered structures. The frequency response curves of the wave passing ratio are shown in Figure 9. The vertical axis of the curve represents the normalized wave passing ratio, while the horizontal axis represents the frequency of the emission wave. The experimental frequency range starts from 4 kHz and ends at 21 kHz, with an increment of 1 kHz. Based on the experimental results shown in the figures, the following conclusions can be drawn:

(1) The frequency response curves of the wave passing ratio exhibit a distinct single peak feature, with the peak occurring around 14 kHz. The varying parameters of pre-compression force, porosity, wire diameter, helix diameter, specimen height, and layered structure do not have a significant influence on the peak frequency. The peak frequency remains consistently within the range of 13 to 15 kHz.
(2) The amplitude of the wave passing ratio at the peak frequency for different parameter settings is reported to be less than 0.18. The value is approximately two to three times the amplitude observed in the frequency range before and after the peak. This result suggests that EMWMs exhibit good wave isolation characteristics and the influence of the single peak on the response appears to be relatively small. The observed trend of the pre-peak frequency range (4 kHz to 13 kHz) has a significantly higher wave passing ratio compared to the post-peak frequency range (15 kHz to 21 kHz), at approximately twice the magnitude. This result indicates that the wave isolation efficiency of EMWMs is better in the high-frequency region compared to the low-frequency region of the acoustic range.
(3) Based on the experimental results of specimens with different parameters, it can be observed that the increase in pre-compression force leads to an increase in wave passing ratio. The increase in wire diameter leads to a decrease in wave passing ratio. The increase in helix diameter leads to a decrease in wave passing ratio. The increase in porosity results in a decrease in wave passing ratio. The increase in sample height leads to a decrease in wave passing ratio. The effect of the layered structure sample on wave passing ratio is not significant. Among them, the parameter of porosity has the greatest influence on the wave propagation capability of EMWMs. An increase in porosity of 15% results in a 27% decrease in the response.

**Figure 10:**
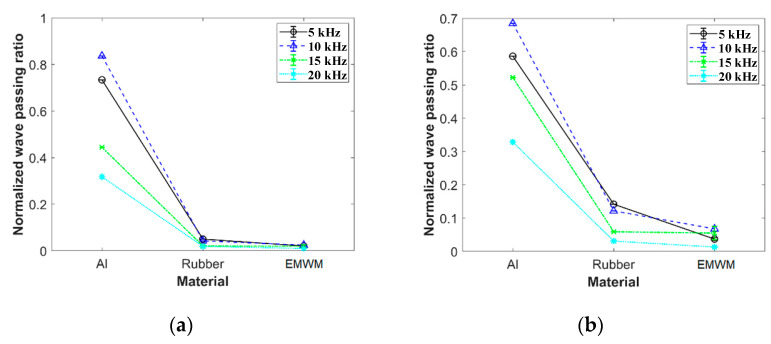

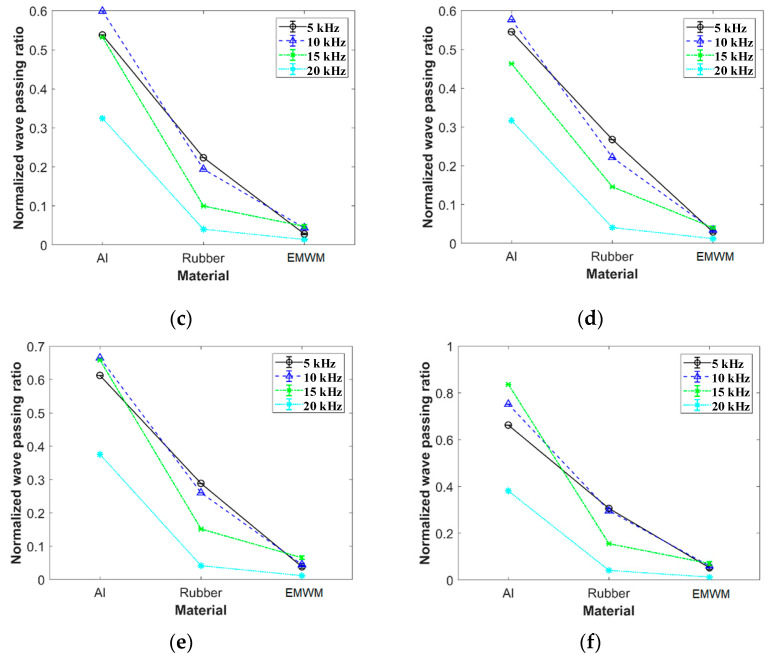
Comparison with wave passing ratio of different materials, with the pre-compression force of (**a**) 50 N, (**b**) 100 N, (**c**) 150 N, (**d**) 200 N, (**e**) 250 N, and (**f**) 300 N.

The experiment also compared the wave passing ratios of an EMWM (porosity 0.77), aluminum alloy, and natural rubber under different pre-compressive force conditions, as shown in Figure 10. Under the same experimental conditions, the wave passing ratio of natural rubber was approximately 2–7 times higher than that of the EMWM, while the wave passing ratio of aluminum alloy was significantly higher than that of the EMWM. The results reflect the excellent acoustic frequency vibration isolation characteristics of the EMWM.

## 5. Numerical Simulation Reproduction

**Figure 11:**
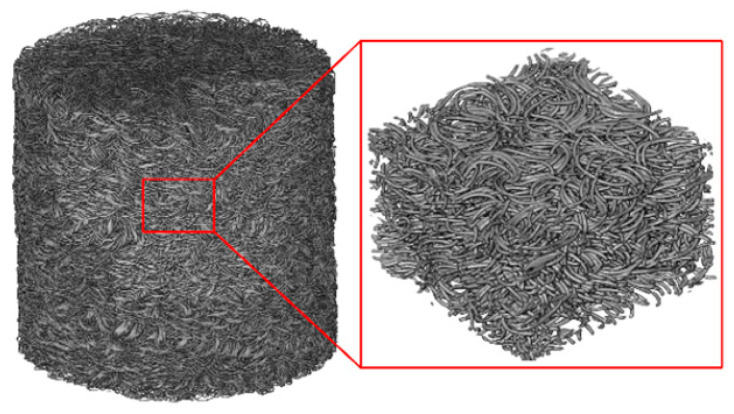
The meso-scale structure of an EMWM obtained by CT scanning.

**Figure 12:**
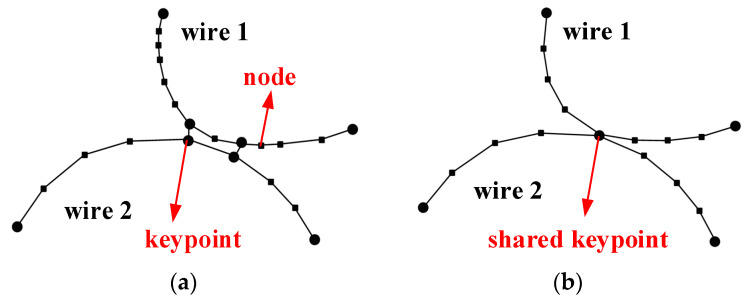
Schematic diagram of contacted wires’ modification in EMWM skeleton model: (**a**) before modification and (**b**) after modification.

For a further understanding of the experimental results, this work utilizes CT scanning and skeleton extraction algorithms to establish the spatial structure of the EMWM specimens, as shown in Figure 11. Firstly, the CT scanning images are reconstructed using the commercial software Avizo v9.0, resulting in a three-dimensional model composed of voxels. Then, the skeleton algorithm is employed to binarize the chromaticity of the three-dimensional model, distinguishing between the wires and gaps. Thereby, both the solid model and the skeleton (the central axis of the wire spirals) are obtained. Then, the skeleton model is imported into Matlab, where the keypoints and nodes of the skeleton are identified for finite element mesh generation. According to the experimental results mentioned in Section 4.1, that the wave passing ratio is not significantly affected by the load amplitude, the contact points between the wires in the skeleton model are merged and set as a shared keypoint, as shown in Figure 12. Finally, the processed skeleton model is imported into the finite element software ANSYS v17.0. The model is divided with two-node BEAM188 beam elements.

**Figure 13:**
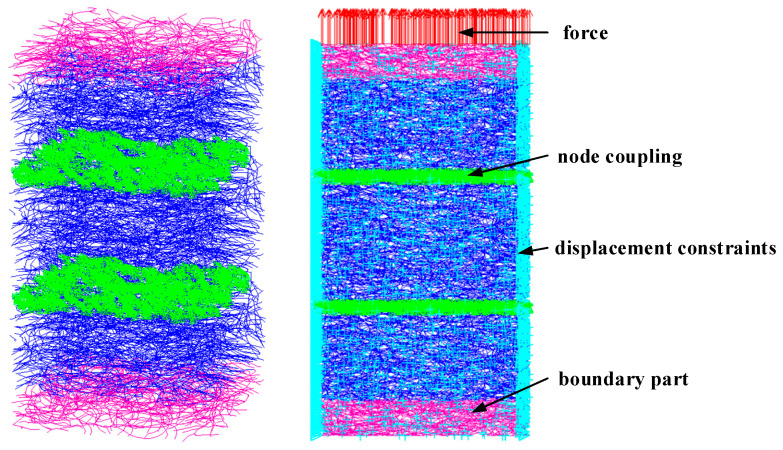
Finite element model and boundary conditions for numerical analysis.

To study the propagation characteristics of one-dimensional compression waves in EMWMs, a representative volume element with dimensions of 6 mm in length, 6 mm in width, and 4 mm in height is selected for simulation in this work. It has been verified that this RVE model can accurately simulate the mechanical characteristics of the EMWM in reference [8]. Considering that the Poisson’s ratio of EMWMs is close to 0, displacement constraint in the normal direction is applied to the sides of the finite element model. A harmonic excitation is applied to the nodes on the upper surface of the model, and harmonic response analysis is conducted. The finite element model and boundary conditions are shown in Figure 13.

The wave passing ratio under different frequencies of harmonic excitation is obtained by harmonic response analysis. The wave passing ratio in the finite element simulation is defined as follows:

(4) $$r_{p,c}=\frac{{\bar{U}}_{down}}{{\bar{U}}_{up}}$$

where ${\bar{U}}_{up}$ and ${\bar{U}}_{down}$ represent the average displacement of the nodes on the upper and lower boundary of the model, respectively.

**Figure 14:**
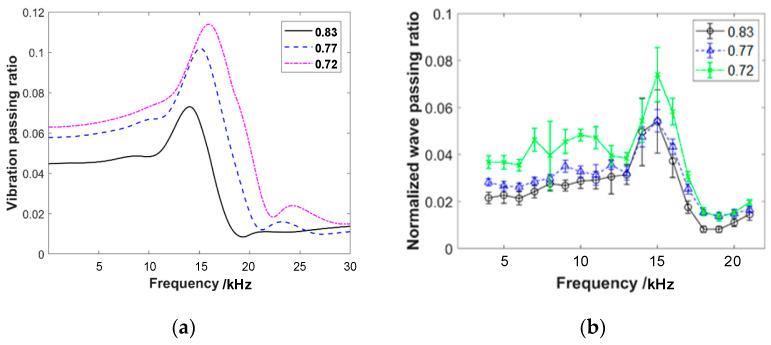
Comparison of wave passing ratio of EMWMs with different porosities: (**a**) simulation results and (**b**) experimental results.

**Figure 15:**
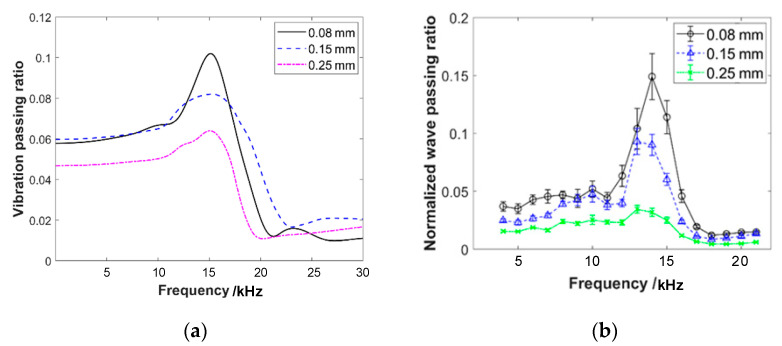
Comparison of wave passing ratio of EMWMs with different wire diameters: (**a**) simulation results and (**b**) experimental results.

**Figure 16:**
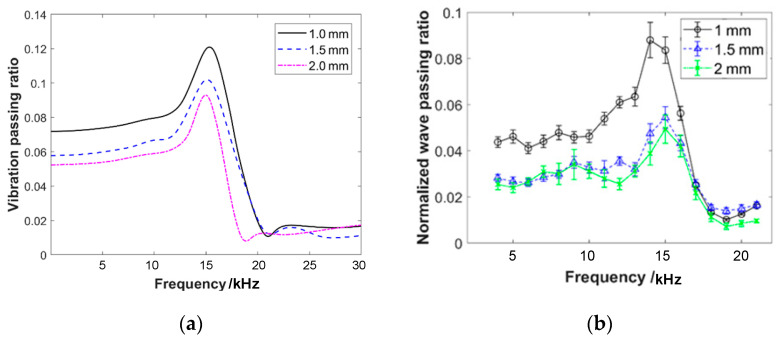
Comparison of wave passing ratio of EMWMs with different helix diameters: (**a**) simulation results and (**b**) experimental results.

**Figure 17:**
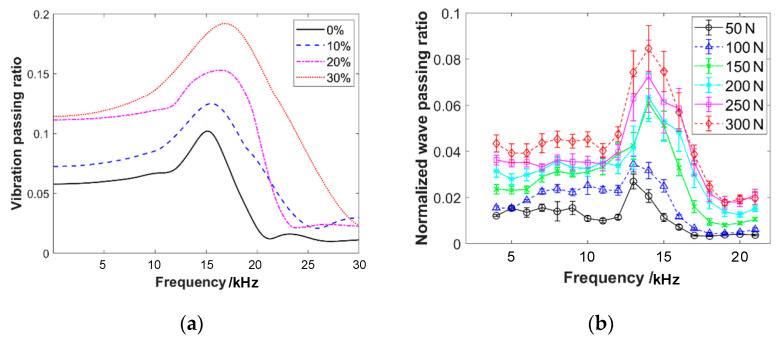
Comparison of wave passing ratio of EMWMs with different pre-compression forces: (**a**) simulation results and (**b**) experimental results.

To reproduce the experimental results, simulations are conducted under the same experimental conditions for different porosity, wire diameter, helix diameter, and pre-compression values. The simulation results for each parameter are presented in Figure 14, Figure 15, Figure 16 and Figure 17. It can be observed that the simulated frequency response curves closely match the experimental results, exhibiting the consistent variation patterns. Although there might be slight differences in value, it indicates that the simulation model proposed in this work effectively explains the experimental phenomena.

## 6. Conclusions

In this work, the acoustic emission setup and a clamping structure are designed for conducting wave propagation experiments. The wave passing ratios of EMWMs are calculated by the time-domain signals of the emission and receiving waves. By varying the frequency, amplitude of the emission waves, and the design parameters of the specimens, the wave propagation characteristics in EMWMs are investigated under different experimental conditions. The main conclusions obtained are as follows:

(1) Under acoustic frequency and small-amplitude excitation, the wave passing ratio of the EMWM is not affected by the amplitude of the waves. It exhibits approximate linear characteristics with a fluctuation coefficient of no more than 15%. It indicates that the contact status between the wires does not affect the mechanical properties of this material.
(2) The frequency response curves of the wave passing ratio in EMWMs exhibit a distinct single peak at around 14 kHz, and the peak values for different parameters are all below 0.18. Compared to aluminum alloy and natural rubber, EMWM is an excellent acoustic frequency vibration isolation material with superior performance.
(3) The wave passing ratio in EMWMs is influenced by design parameters such as pre-compression force, porosity, wire diameter, helix diameter, and specimen height. Among these parameters, porosity has the greatest impact. When applying EMWMs for acoustic frequency vibration isolation, careful consideration of the porosity parameter should be required in the design process.
(4) The finite element model of EMWMs, which merges the contact points between wires, successfully reproduces the frequency response curve characteristics and parameter influences observed in the experiment. However, further research is required to investigate the differences in numerical value between the simulated and experimental results.

## Data Availability

The data presented in this study are available on request from the corresponding author. The data are not publicly available due to privacy.

## Appendix A

The parameters of the EMWM specimens designed for the wave propagation experiment are shown in Table A1. Three specimens were tested in each group.

**Table A1:** The parameters of the EMWM specimens.

Group	Porosity	Wire Diameter /mm	Helix Diameter /mm	Specimen Diameter/mm	Specimen Height/mm
1	0.77	0.08	1.5	20	10
2	0.77	0.15	1.5	20	10
3	0.77	0.25	1.5	20	10
4	0.77	0.08	1.0	20	10
5	0.77	0.08	2.0	20	10
6	0.83	0.08	1.5	20	10
7	0.72	0.08	1.5	20	10
8	0.77	0.08	1.5	20	20
9	0.77	0.08	1.5	20	30
10	0.77	0.08	1.5	20	40
11	0.77	0.08	1.5	20	2
12	0.77	0.08	1.5	20	4
